# Pandemic experiences and psychopathological aspects in individuals with mood disorders and other mental disorders

**DOI:** 10.3389/fpsyt.2022.1039072

**Published:** 2022-10-14

**Authors:** Antonio Del Casale, Carlo Lai, Alessia Iannuccelli, Chiara Ciacchella, Giorgio Veneziani, Mohamed Ali, Clarissa Zocchi, Irene Bilotta, Maurizio Pompili

**Affiliations:** ^1^Department of Dynamic and Clinical Psychology, and Health Studies, Faculty of Medicine and Psychology, Sapienza University of Rome, Rome, Italy; ^2^Unit of Psychiatry, Sant'Andrea University Hospital, Rome, Italy; ^3^Department of Neuroscience, Mental Health, and Sensory Organs (NESMOS), Faculty of Medicine and Psychology, Sapienza University of Rome, Rome, Italy

**Keywords:** mood disorders, COVID-19, skepticism, personality, temperament

## Abstract

Individuals with different personality traits, temperaments, and psychological symptoms have different attitudes toward the pandemic experiences and restrictive measures. The aim of the present study was to evaluate the associations between the psychological factors and the attitudes toward COVID-19, experienced during the third pandemic wave in Italy, in a sample of individuals with psychiatric disorders. Between March and September 2021, 53 patients with mood disorders and other mental disorders completed a survey composed of self-report questionnaires that assessed sleep quality, depressive and hypomanic symptoms, and temperament and personality traits. Positive and negative attitudes toward the pandemic experience were assessed using an *ad hoc* questionnaire. The results showed that individuals with more severe depressive symptoms were less prone to adhere to government guidelines, and were more convinced that the pandemic was not a real problem. Reduced sleep quality was associated with increased skepticism toward official explanations concerning the causes of COVID-19. Lastly, negative affect and cyclothymic temperament predicted the disposition toward COVID-19 vaccines. In conclusion, these findings highlighted that some psychological aspects and psychiatric symptoms could influence the beliefs about COVID-19 and compliance with government recommendations. Further research is needed to provide indications on how to improve the current healthcare policies.

## Introduction

A cluster of atypical pneumonia cases was discovered in Wuhan, China, in December 2019, and the World Health Organization (WHO) described it as Coronavirus disease 2019 (COVID-19) on February 11, 2020 (1). The WHO classified the epidemic as a global pandemic on March 11, 2020, and, by the end of February 2021, there were 110 million confirmed cases and almost 2.5 million deaths globally (2). Governments worldwide adopted necessary restrictive measures to contain the spread of the virus. Beyond the infection itself, the pandemic has resulted in changes in daily life that have contributed to the development of depressive symptoms in the general population, especially among young adults and in countries with more substantial restrictions (3–6). Changes in daily routine schedules have also impacted circadian rhythms, with domestic quarantine determining the loss of external elements that act as synchronizes of the biological clock for the organism (7). As a result, the quantity and quality of sleep were reduced during the pandemic (8, 9). In these circumstances, individuals with pre-existing psychological problems were more likely to experience disease recurrences or symptomatic worsening of disease (4, 10). Some people with bipolar disorder seemed to be particularly affected by economic and job difficulties caused by restrictions, which were linked to psychological discomfort (11) or even post-traumatic stress symptoms (12). Several studies reported that individuals with psychopathologies had worse cognitive symptoms with higher levels of pandemic-related discomfort, anxiety, and sleep difficulties (13, 14). Since the pandemic's beginning, governments have been faced with the skepticism of some people about the COVID-19 phenomenon, which intense doubt affected the management of the health emergency (15). Disbelief in COVID-19 was negatively associated with compliance with government recommendations in general (16) and specifically in taking preventive actions (e.g., social distancing, wearing masks) and intentions to vaccinate (17). In this regard, it was reported that the low perception of the disease risk and the mistrust about the safety and the effectiveness of the vaccination could lead to the refusal or the delay in acceptance of vaccination, despite its availability (18). This resistance toward vaccines was referred to as “vaccination hesitancy” and severely affected the global efforts to stem the pandemic (18). It seems necessary to highlight the factors that influence “vaccination hesitancy,” considering that for long-term management of the virus, vaccines are one of the most successful and cost-effective ways to prevent the disease. Taking together, the negative attitudes toward the pandemic experiences, seemed to be influenced by several psychological factors. In particular, it was found that psychological traits played a relevant role: it was not unexpected that the most agreeable, conscientious, and emotionally stable people were found to be more committed to preventive behaviors, such as respect for the social distancing roles and adhering to vaccination (19, 20). The “tendency to openness” personality trait was linked to both the propensity to adopt preventive behaviors and poor mental health outcomes in a study by Han and colleagues (20) as previously hypothesized by Trobst and colleagues (21), the greater propensity to follow preventive rules could, in this case, pass through an increased perception of risk. However, the results showed a negative adherence to preventive practices (e.g., social distancing) when it comes to the “extroversion” trait (22, 23). More studies on this issue produced similar results (20, 24–27). A recent study discovered that personality traits such as “meanness” (a construct that includes characteristics such as poor empathy, superficial relationships with others, rebellion, the search for excitement, exploitation, and cruelty) and “disinhibition” (i.e., the tendency to show lack of control of impulses, poor planning, and an inability to control one's emotions) are predictors of low endorsement of health behaviors during the pandemic (28). Moreover, a recent study found that individuals who said they didn't believe in the Coronavirus pandemic reported more symptoms of borderline personality disorder (29) and more usage of maladaptive defensive mechanisms (30). Psychiatric illness is another factor associated with uncertainty about vaccines and the assumption of preventive behaviors (31). Interestingly, significant difficulties in adhering to preventive behaviors were found in clinical populations suffering from psychotic, depressive symptoms, and substance use dependence (32–35). In this context, it emerged that cyclothymic and anxious temperaments were associated with a worse capacity of resilience toward the spread of the virus and the restrictions imposed; on the contrary, individuals with hyperthymic temperaments showed good levels of adaptation (36). Moreover, people with anxious, dysthymic, and cyclothymic temperaments showed greater vulnerability to developing psychological distress related to the pandemic (37, 38). According to scientific evidence, individuals with different personality traits, temperaments, and psychological symptoms have a different impact caused by the virus and a different attitude toward the pandemic experiences and restrictive measures (19, 39). The present study aimed to investigate the association between temperamental and personality traits, mood symptoms, sleep disturbance, and the negative/positive attitude toward the pandemic experience in a sample of participants with mental disorders.

## Materials and methods

### Participants

The study was conducted between March and September 2021 at the Center of Personalized Medicine and Service of Personalized Mental Health and Pharmacogenomics, Unit of Psychiatry, Sant'Andrea University Hospital, Sapienza University, Rome. For this study, informed consent was obtained from all participants. All methods were carried out in accordance with relevant guidelines and regulations. Specifically, this study complied with the Declaration of Helsinki adopted by the World Medical Association (WMA) at the 18th WMA General Assembly (Helsinki, Finland, June 1964) and subsequently amended by the 64th WMA General Assembly (Fortaleza, Brazil, October 2013). The local ethical committee of Sapienza (Sant'Andrea University Hospital) approved the present study (prot. N. 6,279/2021). The inclusion criterion was having received a diagnosis of bipolar disorder, major depressive disorder, or other DSM-5 (40) diagnoses (mood disorders, schizophrenia spectrum, obsessive-compulsive spectrum disorders, somatic symptom and related disorders, and personality disorders). Exclusion criteria included minors (<18 years) or those of advanced age (>75 years), concurrent substance use disorders (except nicotine dependence), neurological conditions (epilepsy, major neurocognitive disorders, Parkinson's disease, and Huntington's chorea), and severe acute organic illnesses (major cardiovascular pathologies, uncontrolled diabetes, serious toxic, infectious and metabolic diseases, malignancy, liver failure, and renal failure).

### Measures

Self-report scales were administered to the participants to assess the psychological variables of interest for the present study. The Pittsburgh Sleep Quality Index scale (PSQI) (41, 42) was used to determine sleep quality within the previous month. The 19 questions evaluate the sleep quality, the amount, the status, and the severity of sleep disturbances. The PSQI explores subjective sleep quality, latency, duration, and efficiency, the use of sleep medication, and any deterioration in daily work performance. Higher scores indicate poorer sleep and higher levels of disturbance. The Beck Depression Inventory-II (BDI-II) (43, 44) assessed depressive symptoms. The BDI-II is a 21-question, self-report inventory that provides an overall score of depressive symptom severity over the previous 2 weeks. The Hypomania Checklist scale (HCL-32) (45, 46) assessed hypomanic symptoms. HCL-32 consists of 32 hypomanic symptoms that require yes or no answers. A total score greater or equal to 14 is identified as potentially suffering from bipolar disorder (45). There are two dimensions in the HCL-32: active-related hypomania and irritable/risk-taking hypomania. The Temperament Scale from Memphis, Pisa, Paris, and San Diego-Autoquestionnaire scales (TEMPS-A) (47) assessed temperamental traits. The 110 constituent items inquire about the subject's life-long traits along depressive, cyclothymic, hyperthymic, irritable, and anxious dimensions. The short version of the Dark Triad scale (SD3) (48, 49) and the Personality Inventory for the DSM-5 short version (PID-5-BF) (50, 51) were used to assess personality traits. The SD3 is a 27-item measure yielding scores on narcissism (e.g., “Many group activities tend to be dull without me”), Machiavellianism (e.g., “I like to use clever manipulation to get my way”), and psychopathy (e.g., “Payback needs to be quick and nasty”). Participants responded on a five-point scale from disagree strongly to agree strongly. The PID-5-BF measures five higher-order domains, each represented by five items (Negative Affect; Detachment; Antagonism; Disinhibition; and Psychoticism). Lastly, the attitude toward the pandemic experiences and containment measures were assessed using the following self-administered COVID-19-related questionnaire. The questionnaire consisted of six *ad hoc* items conceived by a group of clinical Psychologists of the Department of Dynamic and Clinical Psychology, and Health Studies. The items were formulated to evaluate frustration due to restrictive measures, skepticism, mistrust, and compliance with government guidelines. The participants rated the following items on a scale from zero to seven. Item 1: “How frustrating do you find the restrictions imposed by the ministerial decree?” (0 = Not at all; 7 = A lot); Item 2: “I feel skepticism about the official explanations for the causes of the virus.” (0 = Not at all; 7 = A lot); Item 3: “I believe that the pandemic is not real.” (0 = Strongly disagree; 7 = Strongly agree); Item 4: “The real reason for the lockdown is not to prevent the spread of the virus.” (0 = Strongly disagree; 7 = Strongly agree); Item 5: “I refuse to adhere to the guidelines imposed by the government, as I believe that the pandemic is not a real problem.” (0=Strongly disagree; 7 = Strongly agree); Item 6: “I am in favor of the use of vaccines against the COVID-19 virus.” (0 = Strongly disagree; 7 = Strongly agree).

### Statistical analysis

Comparisons between diagnostic groups (bipolar disorder I vs. bipolar disorder II vs. major depressive disorder vs. other diagnoses) were performed as follows: one-way analyses of variance (ANOVA) were performed on dimensional variables (age; years of illness; number of prior therapies) and chi-square tests were performed on categorical variables (gender; alcohol and substance use; the presence of organic pathologies; antidepressant drugs, antipsychotic drugs, antiepileptic drugs, lithium, and benzodiazepines prescriptions). Correlations (Pearson's r) were performed between the psychological variables (PSQI; BDI-II; HCL-32; TEMPS-A; SD3; PID-5-BF) and the items of the COVID-19-related questionnaire. Stepwise linear regressions were performed using the scores of the scales assessing the psychological and psychopathological dimensions (PSQI; BDI-II; HCL-32; TEMPS-A; SD3; PID-5-BF) as independent variables, and each score of items 2, 5, and 6 of the COVID-19-related questionnaires as a dependent variable. Power analysis conducted using G^*^Power software (*post-hoc* to compute expected achieved power) with an alpha error probability of 0.05, indicated that the sample was sufficiently powered (minimum value of 1-β = 0.85; maximum value of 1-β = 0.99) to detect acceptable effect sizes for a given measure.

## Results

Our study included 53 consecutively admitted outpatients (27 women and 26 men), of whom 16 met the DSM-5 criteria for bipolar disorder type I, 22 for bipolar disorder type II, eight for major depression, and seven for other psychiatric diagnoses (two with persistent depressive disorder, two with schizophrenia, one with not specified personality disorder, one with obsessive-compulsive disorder, and one with somatic symptom disorder). The sample of 53 participants had a mean age of 46.8 years (SD = 14.95), with an average of years of illness 16.13 (SD = 12.15). Considering that the variable years of illness did not show a normal distribution, we used the Kruskal-Wallis one-way ANOVA among diagnostic groups (bipolar disorder type I vs. bipolar disorder type II vs. major depressive disorder vs. other diagnoses). There were no significant between-group differences in age and number of previous pharmacological treatments. Patients with other diagnoses had fewer years of illness than patients with bipolar disorder I (H = 17.567; *p* = 0.012), and patients with major depression had fewer years of illness than patients with bipolar disorder I (H = 13.656; *p* = 0.04) (Table 1). The chi-squared tests performed among diagnostic groups showed substantial gender differences (χ^2^ = 14.6; *p* = 0.002), with a greater prevalence of men with bipolar disorder I and most women with bipolar disorder II. Moreover, significant differences were found in the prescription of antidepressant drugs (χ^2^ = 14.2; *p* = 0.003), participants diagnosed with major depression and those with other diagnoses reported more antidepressant drug treatments than the other diagnostics groups. There were no significant differences among the groups related to alcohol and substance use (χ^2^ = 4.0; *p* = 0.259), organic pathologies (χ^2^ = 2.1; *p* = 0.554), prescriptions of antipsychotic (χ^2^ = 1.2; *p* = 0.760), antiepileptics drugs (χ^2^ = 8.0; *p* = 0.239), lithium (χ^2^ = 1.3; *p* = 0.731), and benzodiazepines (χ^2^ = 4.8; *p* = 0.189). Table 2 showed the correlation analyses (Pearson's r) between the Pittsburgh Sleep Quality Index scale (PSQI), the Beck Depression Inventory-II (BDI-II), the Hypomania Checklist scale (HCL 32), the Temperament Scale from Memphis, Pisa, Paris, and San Diego Autoquestionnaire (TEMPS-A), the short version of the Dark Triad scale (SD3), the Personality Inventory for the DSM-5 short version (PID-5-BF), and the items of the COVID-19 related questionnaire. The frustration due to the restrictions imposed by the ministerial decree (item 1) was positively correlated with the PSQI, the BDI-II, the active-elated subscale of HCL 32, and the irritable subscale of TEMPS-A. The skepticism about the official explanations for the causes of the virus (item 2) was positively correlated with the PSQI. The belief that the pandemic is not real (item 3) was positively correlated with the BDI-II. The idea that the real reason for the lockdown is not to prevent the spread of the virus (item 4) was positively correlated with the irritable/risk-taking subscale of HCL 32. The refusal to adhere to the guidelines imposed by the government (item 5) positively correlated with the BDI-II. Lastly, the favor of vaccines against the COVID-19 virus (item 6) positively correlated with the total of PID-5-BF and the negative affect subscale of PID-5-BF. Table 3 showed the results of the linear stepwise regressions performed with the psychological variables as predictors of items 2, 5, and 6 of the COVID-19-related questionnaire. The regression model performed on the skepticism about the official explanations for the causes of the virus was significant, with sleep quality as a significant predictor. Moreover, the regression model on adherence to the guidelines showed the BDI-II mean score as a significant predictor. Finally, the regression model on the willingness to use vaccines against COVID-19 showed cyclothymic temperament and negative affect as significant predictors.

**Table 1:** Comparisons (Kruskal-Wallis one-way ANOVAs) between diagnostic groups (bipolar disorder type I vs. bipolar disorder type II vs. major depressive disorder vs. other diagnoses) on dimensional variables (age; years of illness; the number of prior therapies).

	**Bipolar disorder type I**	**Bipolar disorder type II**	**Major depressive disorder**	**Other diagnoses**	**H**	***p***
Age (M ± SD)	51.1 ± 15.7	45.9 ± 12.8	49.7 ± 13.9	36.6 ± 18.4	5.481	0.14
Years of illness (M ± SD)	20.7 ± 12.5	16.8 ± 11.8	11.7 ± 12.9	8.7 ± 7.6	8.54	**0.036**
Number of prior therapies (M ± SD)	3.7 ± 2.4	3.9 ± 2.7	5.1 ± 4.1	1.4 ± 1.3	6.921	0.074

Significant for *p*-value < 0.050 in bold.

**Table 2:** Correlations (Pearson's r) performed between the measures of the psychological variables (the Pittsburgh sleep quality index scale, the Beck depression inventory-II, the hypomania checklist scale, the temperament scale from Memphis, Pisa, Paris, and San Diego-autoquestionnaire, the short version of the dark triad scale, and the personality inventory for the DSM-5 short version) and the items of the COVID-19 related questionnaire.

		**Item 1**	**Item 2**	**Item 3**	**Item 4**	**Item 5**	**Item 6**
PSQI	*r* *p*	**0.33** **0.027**	**0.42** **0.005**	0.23 0.126	0.28 0.064	0.25 0.097	0.08 0.588
BDI-II	*r* *p*	**0.30** **0.042**	0.13 0.393	**0.30** **0.040**	0.19 0.206	**0.38** **0.008**	−0.17 0.270
HCL32 active elated	*r* *p*	−0.13 0.387	−0.19 0.219	−0.20 0.190	−0.04 0.815	−0.25 0.096	−0.01 0.943
HCL32 irritable/risk taking	*r* *p*	**0.38** **0.010**	0.20 0.190	0.10 0.531	**0.38** **0.010**	−0.01 0.944	0.16 0.302
HCL32 total	*r* *p*	0.01 0.940	−0.14 0.373	−0.15 0.316	0.11 0.494	−0.22 0.143	0.02 0.910
TEMPS depressive	*r* *p*	0.09 0.546	0.02 0.870	−0.01 0.937	−0.12 0.417	0.21 0.154	0.08 0.611
TEMPS cyclothymic	*r* *p*	0.03 0.831	0.02 0.893	−0.01 0.930	0.02 0.869	0.18 0.220	−0.20 0.185
TEMPS hypertimic	*r* *p*	0.21 0.164	0.13 0.403	0.04 0.781	0.20 0.185	−0.14 0.356	0.05 0.729
TEMPS irritable	*r* *p*	**0.37** **0.010**	0.13 0.397	0.06 0.708	0.15 0.335	0.07 0.646	0.05 0.758
TEMPS anxious	*r* *p*	0.13 0.384	0.27 0.073	0.07 0.654	0.13 0.400	0.27 0.067	0.08 0.610
PID-5-BF total	*r* *p*	0.10 0.526	0.10 0.521	0.03 0.838	0.05 0.752	0.07 0.666	**0.32** **0.036**
PID-5-BF negative affect	*r* *p*	0.24 0.111	0.19 0.223	0.05 0.734	0.08 0.607	0.00 0.975	**0.43** **0.004**
PID-5-BF detachment	*r* *p*	−0.08 0.593	−0.11 0.461	0.03 0.846	0.07 0.664	0.19 0.205	0.28 0.060
PID-5-BF antagonism	*r* *p*	−0.00 0.975	0.14 0.353	−0.06 0.711	−0.17 0.257	−0.20 0.189	0.25 0.101
PID-5-BF disinhibition	*r* *p*	0.14 0.378	0.11 0.457	−0.01 0.940	0.09 0.560	−0.00 0.978	0.05 0.745
PID-5-BF psychoticism	*r* *p*	0.05 0.751	0.05 0.755	0.03 0.863	0.06 0.697	0.16 0.310	0.06 0.715
SD3 machiavellianism	*r* *p*	0.22 0.141	0.14 0.364	−0.05 0.740	0.06 0.694	−0.01 0.944	0.25 0.098
SD3 narcissism	*r* *p*	0.09 0.569	0.14 0.343	−0.05 0.722	0.04 0.810	−0.08 0.587	−0.09 0.534
SD3 psychopathy	*r* *p*	0.07 0.628	0.15 0.319	0.06 0.696	0.10 0.517	0.03 0.836	−0.10 0.518

Significant for *p*-value < 0.050 in bold.

Item 1, How frustrating do you find the restrictions imposed by the ministerial decree?; Item 2, I feel skepticism about the official explanations for the causes of the virus; Item 3, I believe that the pandemic is not absolute; Item 4, The real reason for the lockdown is not to prevent the spread of the virus; Item 5, I refuse to adhere to the guidelines imposed by the government, as I believe that the pandemic is not a real problem; Item 6, I am in favor of the use of vaccines against the COVID-19 virus. BDI-II, The Beck Depression Inventory-II; HCL-32, The Hypomania Checklist scale; PID-5-BF, Personality Inventory for the DSM-5 short version; PSQI, The Pittsburgh Sleep Quality Index scale; SD3, The short version of the Dark Triad scale; TEMPS-A, The Temperament Scale from Memphis, Pisa, Paris, and San Diego-Auto questionnaire.

**Table 3:** Linear stepwise regressions performed with the psychological variables (PSQI; BDI-II; HCL-32; TEMPS-A; SD3; PID-5-BF) as predictors of the score of the items 2, 5 and 6 of the COVID-19 related questionnaire.

Item 2, I feel skepticism about the official explanations for the causes of the virus. R = 0.41; R^**2**^ = 0.17; Adjusted R^**2**^ = 0.15; F (1, 40) = 8.2; p = 0.007; Std. Error of estimate =2.08.					
**Included variable in step 1**	**Beta**	**B**	**Std. Err. of B**	***t***	***p***
PSQI	0.41	0.32	0.11	2.87	0.007
**Excluded variables**	**Beta**	**Partial correlation**	**Collinearity**	**t**	**p**
BDI-II	−0.185	−0.158	0.607	−1.002	0.322
HCL32 active elated	−0.062	−0.066	0.932	−0.410	0.684
HCL32 irritable/risk taking	0.040	0.036	0.673	0.227	0.821
HCL32 total	−0.099	−0.108	1.000	−0.681	0.500
TEMPS depressive	−0.107	−0.112	0.895	−0.702	0.487
TEMPS cyclothymic	−0.079	−0.083	0.911	−0.519	0.607
TEMPS hypertimic	0.015	0.017	0.978	0.103	0.918
TEMPS irritable	−0.019	−0.019	0.819	−0.117	0.908
TEMPS anxious	0.068	0.060	0.628	0.372	0.712
PID-5-BF total	−0.015	−0.016	0.957	−0.100	0.921
PID-5-BF negative affect	0.073	0.078	0.946	0.490	0.627
PID-5-BF detachment	−0.167	−0.182	0.980	−1.155	0.255
PID-5-BF antagonism	0.055	0.060	0.983	0.374	0.711
PID-5-BF Disinhibition	0.052	0.057	0.995	0.358	0.722
PID-5-BF psychoticism	−0.041	−0.044	0.981	−0.278	0.783
SD3 machiavellianism	−0.025	−0.026	0.959	−0.165	0.870
SD3 narcissism	0.058	0.064	1.000	0.402	0.690
SD3 psychopathy	0.030	0.033	0.995	0.204	0.840
Item 5, I refuse to adhere to the guidelines imposed by the government, as I believe that the pandemic is not a real problem. R = 0.39; R^2^ = 0.15; Adjusted R^2^ = 0.13; F (1, 40) = 7.19; *p* = 0.011; Std. Error of estimate =2.29.					
**Included variable in Step 1**	**Beta**	**B**	**Std. Err. of B**	***t***	***p***
BDI-II	0.39	0.07	0.03	2.68	0.011
Excluded variables	Beta	Partial correlation	Collinearity	t	p
PSQI	0.012	0.010	0.607	0.065	0.949
HCL32 active elated	−0.126	−0.128	0.887	−0.809	0.423
HCL32 irritable/risk taking	−0.220	−0.208	0.760	−1.330	0.191
HCL32 Total	−0.175	−0.189	0.992	−1.203	0.236
TEMPS depressive	0.003	0.002	0.649	0.014	0.989
TEMPS cyclothymic	−0.014	−0.013	0.718	−0.082	0.935
TEMPS hypertimic	−0.153	−0.166	0.995	−1.050	0.300
TEMPS irritable	−0.305	−0.251	0.573	−1.619	0.114
TEMPS anxious	0.111	0.104	0.746	0.652	0.518
PID-5-BF total	−0.068	−0.069	0.887	−0.433	0.668
PID-5-BF negative affect	−0.091	−0.095	0.934	−0.597	0.554
PID-5-BF detachment	0.101	0.106	0.945	0.668	0.508
PID-5-BF antagonism	−0.250	−0.269	0.984	−1.745	0.089
PID-5-BF disinhibition	−0.142	−0.145	0.889	−0.918	0.364
PID-5-BF psychoticism	0.070	0.074	0.944	0.462	0.647
SD3 machiavellianism	−0.071	−0.074	0.940	−0.465	0.644
SD3 narcissism	−0.124	−0.133	0.969	−0.839	0.407
SD3 psychopathy	0.006	0.007	0.982	0.041	0.967
Item 6, I am in favor of the use of vaccines against the COVID-19 virus. R = 0.42; R^**2**^ = 0.18; Adjusted R^**2**^ = 0.16; F (1, 40) = 8.72; ***p*** = 0.005; Std. Error of estimate =2.09.					
**Included variable in Step 1**	**Beta**	**B**	**Std. Err. of B**	***t***	***p***
PID-5-BF negative affect	0.42	1.26	0.43	2.95	0.005
Item 6, I am in favor of the use of vaccines against the COVID-19 virus. R = 0.57; R^2^ = 0.33; Adjusted R^2^ = 0.30; F (2, 39) = 9.65; *p* < 0.001; Std. Error of estimate =1.91.					
**Included variable in Step 2**	**Beta**	**B**	**Std. Err. of B**	***t***	***p***
TEMPS cyclothymic	−0.43	−3.83	1.29	−2.98	0.005
PID-5-BF negative affect	0.60	1.77	0.43	4.16	<0.001
Excluded variables	Beta	Partial correlation	Collinearity	*t*	*p*
PSQI	0.126	0.146	0.896	0.908	0.370
BDI-II	−0.032	−0.033	0.716	−0.202	0.841
HCL32 active elated	−0.116	−0.141	0.978	−0.875	0.387
HCL32 irritable/risk taking	0.052	0.057	0.801	0.355	0.725
HCL32 total	−0.092	−0.109	0.930	−0.676	0.503
TEMPS depressive	0.075	0.070	0.591	0.436	0.666
TEMPS hypertimic	0.045	0.054	0.966	0.331	0.742
TEMPS irritable	0.272	0.246	0.546	1.565	0.126
TEMPS anxious	0.220	0.203	0.569	1.277	0.209
PID-5-BF total	0.191	0.127	0.295	0.788	0.435
PID-5-BF detachment	0.119	0.124	0.730	0.772	0.445
PID-5-BF antagonism	0.194	0.225	0.904	1.423	0.163
PID-5-BF disinhibition	0.065	0.070	0.774	0.433	0.668
PID-5-BF psychoticism	−0.138	−0.122	0.524	−0.758	0.453
SD3 machiavellianism	0.203	0.241	0.941	1.533	0.133
SD3 narcissism	−0.037	−0.043	0.919	−0.267	0.791
SD3 psychopathy	0.021	0.025	0.939	0.154	0.879

BDI-II, The Beck Depression Inventory-II; PID-5-BF, Personality Inventory for the DSM-5 short version; PSQI, The Pittsburgh Sleep Quality Index scale; TEMPS-A, The Temperament Scale from Memphis, Pisa, Paris, and San Diego-Auto questionnaire.

## Discussion

The main finding of the present study was that the psychological dimensions were associated with the attitude toward the pandemic experience and the containment measures. Specifically, individuals who exhibited more depressive symptoms were more prone to refuse to adhere to government-imposed guidelines, believing that the pandemic was not a real problem. This result offered a reflection on several implications. On the one hand, it has been shown that the increase in the restrictions imposed corresponded to a rise in the general population of depressive symptoms (5). On the other hand, it was also known that more significant psychological distress was not reflected in greater adherence to restrictions (52). The studies carried out during the second pandemic wave (from October 2020 to January 2021) showed how the stress that lasted from March 2020 resulted in an increase in depressive symptoms and lower adherence to preventive behaviors during the second phase of restrictions (53, 54). Therefore, it was conceivable that this trend also characterizes the third wave (from March 2021 to June 2021), and people who were the most vulnerable to developing depressive symptoms became impatient with government regulations. Such data offered insights into the management of the pandemic emergency. As shared widely, failing to act under the containment rules represents one of the primary limits in combating viral spread. Therefore, it could go into the argument with further studies, verifying whether treating the underlying mood disorder cannot improve adherence to government rules in individuals with mood disorders. Moreover, the item used in the study, “I refuse to adhere to the guidelines imposed by the government, as I believe that the pandemic is not a real problem,” offered a different investigation perspective. The attitude of believing that the pandemic problem has been overestimated and that excessive precautions have been taken creates typical of the so-called “COVID Disregard Syndrome,” which has been seen to be associated with reluctance toward preventive measures, anti-vaccine tendencies, and the phenomenon of the psychological reactance that arises from the perception where the imposed rules constitute an attack on free will (55). Therefore, individuals with these characteristics could be more affected by the psychological distress induced by the pandemic. Furthermore, the statement “I believe that the pandemic is not a real problem” it could also be read more concretely as an expression of conspiracy theories. In this case, in line with the most recent scientific literature, it would confirm that those who adhere to conspiracy theories could be more susceptible to emotional distress and less inclined to comply with public health regulations during the pandemic (49, 56). Another result of the present study was that sleep problems were associated with an increased skepticism toward official explanations concerning the causes of COVID-19. Uncertainty, psychological distress, paranoid thinking, and anxiety, all factors that predispose to sleep disturbances, are common in individuals who embrace conspiracy theories (57, 58). Furthermore, it was found that personality traits and cyclothymic temperament predicted the disposition toward COVID-19 vaccines. A broader favor emerged from those who expressed more dimensions of “negative affect,” which was examined as aforementioned according to the PID-5-BF scale. This finding is in line with the current scientific literature, where it has emerged that individuals with these characteristics showed more propensity to respect the social distance and apply proper hand hygiene (22). Completing the vaccination is considered a prosocial attitude in the context of the pandemic. Furthermore, high scores in the negative affect domain were predictive of anxious symptoms in the pandemic context (49, 59). Therefore, it would be necessary to further research if the propensity to get vaccinated could also be explained by more significant anxiety due to the contagion. Instead, an opposite attitude emerged toward anti-COVID vaccines in study participants characterized by a cyclothymic temperament. In this regard, it has been reported previously that individuals with cyclothymic temperaments tend to exhibit a tendency toward overoptimism and a high propensity for high-risk behaviors (60). It could be hypothesized that these characteristics could lead these individuals to underestimate adherence to medical recommendations, such as vaccines. However, it will be necessary to identify additional factors that explain this highly complex phenomenon. The results of this study should be taken with caution, considering that the main limitation is the low number of participants. However, for regression analyzes in the medical setting, a number of participants >25 is considered sufficient to reduce possible bias due to sample paucity (61). Another limitation could be related to social desirability, which could have affected the answers to the self-report measures used in this study. Future research should be implemented with larger samples to study these under-investigated phenomena. In conclusion, the present study highlighted the psychological factors associated with the experiences and attitudes toward the COVID-19 pandemic in a sample of individuals with psychiatric disorders during the third pandemic wave in Italy. These results offered insights into the need to improve the current healthcare policies: planning adequate treatments for the improvements of psychiatric symptoms could promote a better attitude toward the COVID-19 experience and greater adherence to containment measures. In this regard, the healthcare system needs to change rapidly to cope with the difficulties associated with the pandemic by refining current practices. The results of the present study suggest that the implementation of *ad hoc* health policies and individualized interventions that take into account the temperament and personality traits of individuals with psychiatric disorders could promote better infection risk management for this vulnerable group.

## Data availability statement

The raw data supporting the conclusions of this article will be made available by the authors, without undue reservation.

## Ethics statement

The studies involving human participants were reviewed and approved by Local Ethical Committee of Sapienza (Sant'Andrea University Hospital). The patients/participants provided their written informed consent to participate in this study.

## Author contributions

AD, AI, CZ, IB, and MP conceptualized and designed the study. AD and AI analyzed the data. AD, CL, AI, CC, GV, and MA participated in drafting the manuscript. All authors contributed to the interpretation of the results, in the critical revision of the manuscript, and approved the current version as the final manuscript.

## Conflict of interest

The authors declare that the research was conducted in the absence of any commercial or financial relationships that could be construed as a potential conflict of interest.

## Publisher's note

All claims expressed in this article are solely those of the authors and do not necessarily represent those of their affiliated organizations, or those of the publisher, the editors and the reviewers. Any product that may be evaluated in this article, or claim that may be made by its manufacturer, is not guaranteed or endorsed by the publisher.
